# The Studies on Gallium Nitride-Based Materials: A Bibliometric Analysis

**DOI:** 10.3390/ma16010401

**Published:** 2023-01-01

**Authors:** Weng Hoe Lam, Weng Siew Lam, Pei Fun Lee

**Affiliations:** Department of Physical and Mathematical Science, Faculty of Science, Universiti Tunku Abdul Rahman, Kampar Campus, Jalan Universiti, Bandar Barat, Kampar 31900, Perak, Malaysia

**Keywords:** gallium nitride, semiconductor, bibliometric analysis, subject area, VOSviewer

## Abstract

Gallium nitride (GaN) has a wide energy band gap and a high power density, efficiency, switching frequency, and electron carrier mobility, having broad applications in digitization. Because GaN has high potentials, this study performed a bibliometric analysis on the publications of GaN indexed in the Web of Science database from 1970 to 2023. A performance analysis of the 15,634 publications was performed using Harzing’s Publish or Perish tool, while science mappings were performed with VOSviewer software. The results show that there has been an uptrend in the on-going research on GaN, especially in the past decade. Most of the documents are within the fields of physics, engineering, and materials science. The United States has the highest number of publications and the most impactful research. The United States is also actively collaborating with other countries to gain deeper insights into GaN. The analysis shows that the concentration of GaN research is slowly moving towards the development of high-voltage operations.

## 1. Introduction

From germanium and silicon to gallium arsenide [1,2,3], researchers and practitioners in the semiconductor industry are constantly looking for materials with greater power density and efficiency, especially for automation, artificial intelligence, Internet of Things, and 5G technology [4,5]. Even though silicon is inexpensive and abundantly available, it is more suitable for technologies with lower frequencies. The performance of the silicon will drop as the temperature rises [6]. This is a serious problem, as current and future integrated circuits are more complex and will likely have high heat generation. Silicon also has a lower electron mobility compared with other materials such as the III-V semiconductors [7]. For high frequencies, gallium arsenide has been a popular option for manufacturers. However, gallium arsenide is highly brittle as the interatomic bonds can easily break. Cracks usually form during the nano-cutting phase as the cutting depth increases. The dicing of gallium arsenide wafers will also cause fracture, which is costly to manufacturers [2,8].

Overall, the industry has recognized silicon as the initial generation semiconductor and gallium arsenide as being the next generation semiconductor [9]. In Industry 4.0, the current wave has focused on semiconductors with wide energy bandgaps of above 2.3 eV, with gallium nitride (GaN) and silicon carbide (SiC) being the most prominent semiconductors [10]. As the latest generation of semiconductors, GaN and SiC have higher power densities and efficiencies compared with the first and second generations. The advantages of the current generation semiconductors include small gate capacitance, gate drive loss, and output capacitance while also having a high switching frequency [11].

Even though GaN and SiC are the modern-day semiconductors, there are some differences between them. As a field-effect transistor, GaN does not have body diode. Therefore, GaN does not have reverse recovery loss [12]. The switching energy is also lower in GaN compared with SiC, which means GaN has smaller loss in the power factor correction (PFC) [13]. The switching speed of GaN can also reach 150 V/ns, which indicates a higher efficiency [11,14]. There is also smaller dead-time loss in GaN than in SiC. The adoption of GaN is also relatively cheap as it does not require a high number of active and passive components [11]. The cooling capability of GaN is also excellent, which reduces the need for cooling in a system [15]. Moreover, GaN has a higher electron saturated drift velocity of about 2.20 × 10^7^ cm/s and electron mobility of 990–2000 cm^2^/V_s_ as compared with SiC, which is 650 cm^2^/V_s_ [15,16]. In short, GaN is excellent for many applications in systems with low temperatures and high frequencies as GaN has great figures of merit (FOMs) [17].

In 1875, Paul-Émile Lecoq de Boisbaudran, a French chemist, discovered gallium in a sample of mineral sphalerite. De Boisbaudran performed a test using spectroscopy and found a pair of violet lines which signaled eka-aluminium. The earliest pure gallium was then collected through electrolysis, with the measured density of 5.9 g ml^−1^ [18]. GaN was then produced through the reaction of metallic gallium with ammonia gas at around 1000 °C in the 1930s [19,20]. The first concept of the light emitting diode (LED) was demonstrated by Maruska et al. [21] in 1973 where a high voltage of about 160 V was needed to obtain the violet luminescence. Currently, GaN-based LEDs, with sizes of smaller than 100μm, have high current density, high efficiency in generating blue and green light, and high modulation bandwidth, which are highly suitable for optical wireless communication [22,23].

GaN is hard and has a strong chemical stability and a melting point reaching up to 1700 °C [24]. It also has a wide bandgap of 3.4 eV and a hexagonal P63mc wurtzite crystal structure at atmospheric pressure [25,26]. GaN has been widely applied in 5G technologies [25]. Due to the high chemical stability and wide bandgap, GaN is resistant to radiation, allowing signals to be steady and accurate despite disturbance [27]. Low gate charge and high frequency, which bring the loss of efficiency to a minimum, also help in speeding up the switching rate for faster calculation in 5G technologies [28,29]. The high thermal conductivity of 2.0 Wm^−1^K^−1^ and good heat dissipation imply that GaN is suitable to be used in 5G base stations [27]. In the future, with the mass production and wide application, the cost of using GaN can be reduced as GaN becomes an important material in the industry.

A bibliometric analysis examines the scientific performances of a specific topic in a scholarly database [30]. Bibliometric analyses are important for studying the impacts of scientific publications in terms of citation metrics, subject areas, geographical regions, keywords, and authorships [31]. This type of analysis also helps to uncover the various domains in a specific topic [32]. The outcome of a bibliometric analysis helps scholars to identify emerging trends in the selected topic and the research gaps which could be further explored for a more comprehensive coverage of the topic [33]. For an in-depth understanding of the topic, a bibliometric analysis covers two important parts, which are performance analysis and scientific mapping [34,35].

A performance analysis involves the use of citation metrics such as citation counts, citation impacts, *h*-indexes, and *g*-indexes [36,37]. Citation counts include the total number of citations from a set of publication, such as the total citation (TC) received from the publications in a particular year; citation impacts study the average citations per paper (C/P) or the citations per cited paper (C/CP). The *h*-index means the “*h*” number of publications that has received “*h*” number of citations, which is used to assess the quality of a research achievement; the *g*-index involves the “*g*” number of publications, whereby the average citation is “*g*^2^” and above [38,39,40]. In short, a researcher shall receive an *h*-index if the *h* of the researcher’s total number of papers (*N*) has been cited at least *h* times while the remaining papers (*N-h*) do not have greater than *h* citations, respectively. A high *h*-index shows that a researcher has consistently produced a high number of impactful papers [41]. When a set of publications is ranked in descending order of the number of citations obtained, the *g*-index shows the largest number of the top *g* papers that received at least *g*^2^ citations together. The *g*-index is different from *h*-index, where a high *h*-index requires a high number of quality publications. However, a high *g*-index can be attributed to only a small number of papers [42,43,44].

Meanwhile, scientific mapping shows the knowledge dynamics in the topic. Scientific mapping shows the collaborative networks of authors and relationships among the keywords [45,46]. For a performance analysis, Harzing’s Publish or Perish tool is used [47,48,49,50]. VOSviewer is a popular open-source program for scientific mapping, especially for network and density visualizations [51,52]. To the best of our knowledge, there has been no bibliometric analysis study performed on GaN from the first indexed paper in 1970 to the latest publications in the Web of Science database. Therefore, the aim of this study was to perform a bibliometric analysis on GaN from 1970 to 2023 using the Web of Science database. To date, Web of Science has more than 21,100 peer-reviewed publications, which are of high quality. Therefore, it is highly suited for a bibliometric analysis [30,53,54]. This paper shall continue with the historical development and applications of GaN, the data and methodology, the results and discussions, and the conclusion in the following sections.

## 2. Historical Development and Applications of Gallium Nitride

Roccaforte and Leszczynski [19] summarized the historical development of nitrides research. The important historical steps are shown as follows.

1932: The first polycrystalline GaN material was synthesized by flowing ammonia (NH_3_) over liquid gallium (Ga) at around 1000 °C [20].

1938: The crystal structure of GaN has been studied in GaN powders [55].

1969–1971: Thin GaN layers were grown by Maruska and Tietjen [56] using hydride vapor phase epitaxy (HVPE) on sapphire substrates.

1972: Manasevit et al. [57] and Manasevit [58] grew the first metal–organic vapor-phase epitaxy (MOVPE) GaN layers.

1990: Matsuoka et al. [59] succeeded in the growth of the first InGaN layers, offering access to a very wide spectral range from 0.7 eV (IR) to 3.5 eV (UV) through all wavelengths of the visible range.

2001: Sumitomo Electric bought the patent from Tokyo Agriculture University on the DEEP method to grow GaN single crystals on GaAs substrates using the HVPE method and bowing dislocations in small regions [60,61].

2014: The Nobel Prize in Physics was assigned to three Japanese scientists (Isamu Akasaki, Hiroshi Amano, and Shuji Nakamura) for the invention of efficient blue LEDs, which has enabled bright and energy-saving white light sources [62,63].

2019: Zhang et al. [64] demonstrated 271.8 nm laser diodes (LDs) operating at room temperature and in the pulse mode.

There are several applications of GaN-based materials in optoelectronic devices. The nitride-based optoelectronic devices such as LEDs and LDs are applied in lighting, communications, and quantum applications [65]. GaN LDs allow data speeds reaching 15 Gbit/s with the combination of orthogonal frequency division multiplexing [65]. GaN LDs can also be applied in areas of high spectral purity, such as atom cooling and optical reading or opto-magnetic memories [66]. White LEDs have significantly decreased the energy consumption and increased the contrast ratio and efficiency with a luminous efficacy of more than 150 lm W^−1^ [22,67]. White LEDs are constructed using blue LEDs illuminating phosphor to excite light with longer wavelengths [68,69]. Such white GaN LEDs are used as bulbs or headlights and in computer screens [19]. GaN LEDs also contribute to smart lighting, which includes those used for tracking and imaging, which is a part of optical wireless communication [23]. Moreover, GaN is also found to be promising for power conversion applications in aerospace because of the higher slew rate, low ON resistance, and small die size [70]. Satellites with GaN solid-state power amplifiers were also launched more than six years ago by BeiDou Navigation Satellites and are still currently adopted. This power amplifier has an output power of greater than 150 W and a greater than 50% efficiency [71].

## 3. Data and Methodology

**Figure 1:**
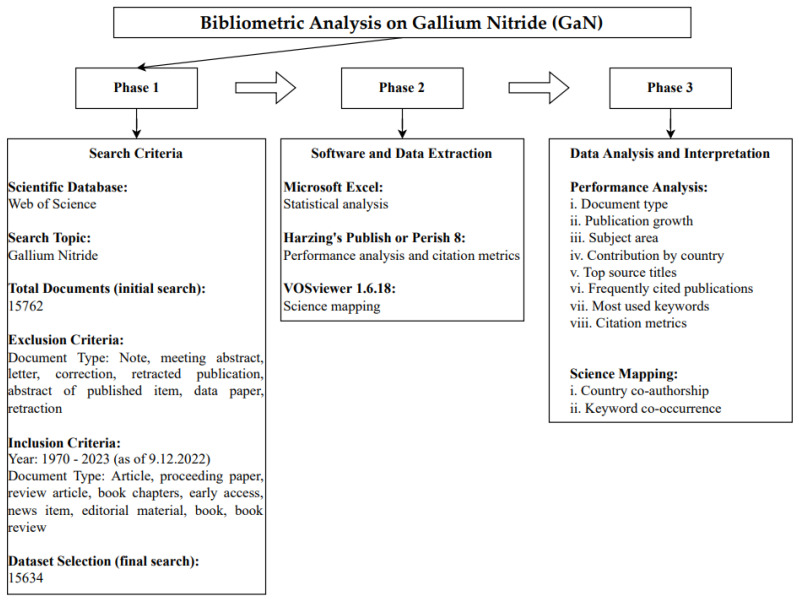
The three-phase approach for the bibliometric analysis on GaN.

This paper performs a bibliometric analysis on GaN publications indexed in the Web of Science database [72,73]. This study adopts a three-phase approach that includes: (1) search query identification, (2) software and data extraction, and (3) data analysis, as presented in Figure 1 [74].

For the initial phase, the topic of study, “Gallium nitride”, was first identified for the bibliometric analysis. Scientific literature on GaN was then searched on the Web of Science database because of its wide coverage and high-quality peer-reviewed papers, which also allows for bibliographic information extraction [75]. Data were collected on 9 December 2022 with the following query: (“gallium nitride”(Topic)), which yielded 15,762 documents. After that, the articles, proceeding papers, review articles, book chapters, early access, news item, editorial material, books, and book reviews were included [74,76]. The final dataset consists of 15,634 documents ranging from 1970 to 2023.

In the second phase, the data were exported in the plain text file format for the statistical analysis of bibliometric information such as years, author names, subject areas, document types, source titles, keywords, and countries. After that, intensive citation analysis using Harzing’s Publish or Perish 8 and bibliometric mapping using VOSviewer version 1.6.18 were performed [77,78]. The performance analyses of the TC, C/P, C/CP, *h*-index, and *g*-index according to year, country, and source title were obtained with Harzing’s Publish or Perish [49]. Then, VOSviewer was used for science mapping, including country co-authorship and keyword co-occurrence analyses [79].

## 4. Results

This section contains the results of the bibliometric analysis on GaN from 1970 to 2023 as of 9 December 2022. Table 1 presents the breakdown of the document types of the documents on GaN. These documents are composed of articles (11,559 documents or 67.16%), proceeding papers (5112 documents or 29.70%), review articles (311 documents or 1.81%), book chapters (86 documents or 0.50%), early access documents (59 documents or 0.34%), news items (40 documents or 0.23%), editorial materials (39 documents or 0.23%), books (5 documents or 0.03%) and book reviews (1 document or 0.01%). By comparison, articles and proceeding papers make up more than 96% of the total documents [80].

### 4.1. Production Growth

Table 2 shows the annual production growth of GaN documents from 1970 to 2023 as of 9 December 2022. Because the initial publications are in 1970, the total annual publication until 1990 has been very low. Based on our search query that covers all topics of GaN, including title, abstract, author keywords, and Keywords Plus, the first three papers indexed in Web of Science were published in 1970. The first paper by Manchon et al. [81] performed optical studies of the photons and electrons in GaN using first-order Raman spectroscopy and infrared reflectivity. This paper received 156 citations as of 9 December 2022. The second paper was published by Isherwood and Wickenden [82] in 1970, which received 15 citations; this paper investigated the preparation of GaN from gallium arsenide (GaAs). GaN was first noticed at 750 °C when GaAs was nitrided in 50% ammonia–nitrogen gas. The temperature between 750 °C and 870 °C was optimal for the formation of single-phase bulk GaN. The third paper was published by Faulkner et al. [83] in 1970. The researchers investigated the preparation of thin-film GaN by the reaction between gallium trichloride and ammonia.

However, according to the Scopus database, the first indexed paper was published by Margrave [84] in 1956, which found that GaN vaporizes to become complex gaseous nitride at 900–1000 °C. After that, there were two papers published in 1965 and listed in the Scopus database. The first paper in 1965 is “Activation energy for the sublimation of gallium nitride” by Munir and Searcy [85], which has been cited 120 times. This paper intended to study the thermal stability in the form of the vaporization of GaN. The authors noted that even though gaseous nitride was not observed, it can be found that GaN had a high enthalpy of activation for sublimation. Another paper published in 1965, “Vaporization catalysis. The decomposition of gallium nitride” by Schoonmaker et al. [86] received 69 citations as of 9 December 2022. In this paper, the researchers found that GaN has a low vaporization coefficient because of its strong covalent bonds in the rigid wurtzite crystalline structure, therefore it requires a high activation energy. This paper also suggested that metallic gallium or indium has the ability to enhance the vaporization catalysis of GaN.

From 1991 to 1999, there has been an increase in the number of papers, from 16 documents in 1991 to 426 documents in 1999. Even though there were fluctuations in the number of publications from 2000 to 2009, the number of publications increased from 2010 (with 416 publications) and the number of publications peaked in 2020 with 1177 documents. There was a slight drop in the number of papers in 2021 with 1091 publications. Even though there are only 740 publications listed in 2022, 17 papers have been published for 2023 as of 9 December 2022, which clearly show there is ongoing research on GaN.

**Figure 2:**
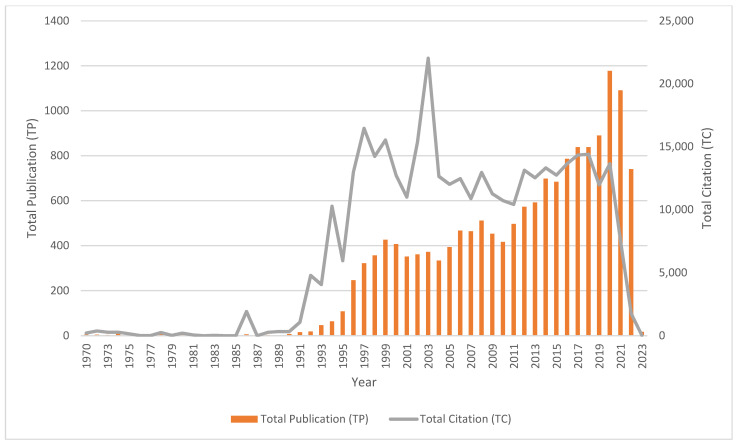
Production and citation trends of GaN documents.

Table 2 shows the citation metrics with regards to the annual production of GaN documents. Total publication (TP) refers to the number of published papers that are indexed in the particular year. Total citation (TC) explains the total number of times the publication has been cited by other papers. Out of the total publications (TP) in a year, the number of papers that have been cited by other papers is reflected by the number of cited papers (NCP). The maximum total citation (TC) of 22,034 citations was recorded for 2003. This was mostly contributed by the top one and top seven cited documents, titled “One-dimensional nanostructures: synthesis, characterization, and applications” by Xia et al. [87] with 8010 citations and “Band parameters for nitrogen-containing semiconductors” by Vurgaftman and Meyer [88] with 2312 citations. The production and citation trends of GaN documents were also described in Figure 2. For citation impact, the highest citation per paper (C/P) and citation per cited paper (C/CP) were recorded in 1989 with 332 citations per paper and 332 citations per cited paper. This is because there were 332 citations from only one total publication (TP) in 1989. The paper titled “Growth of cubic phase gallium nitride by modified molecular-beam epitaxy” by Paisley et al. [89] received 332 citations as of 9 December 2022. The highest *h*-index (*h*) of 58 was recorded for 2000. This means that there were 58 documents that have received at least 58 citations. The highest *g*-index (*g*) of 131 was recorded for 2003. This implies that there are 131 documents with at least 17,161 citations in 2003.

### 4.2. Subject Area

The 15,634 publications have been categorized into various subject areas. Most of the GaN publications are under physics (8250), engineering (6634), and materials science (5197). GaN is also related to chemistry (1607), science technology and other topics (1528), optics (1337), crystallography (827), telecommunications (805), computer science (762), and energy fuels (716). The top 20 subject areas are tabulated in Table 3.

### 4.3. Contribution by Country

Researchers from more than 100 countries have contributed to the literature of GaN from 1970 to 2023 as of 9 December 2022. The top three countries with the highest TP are the United States (4685), China (2808), and Japan (1531). Among the 373,603 total citations received from all 15,634 documents, the United States received 158,750 citations, which was more than 42% of the total citations. Documents from researchers in the United States were also the most impactful, as the documents have the highest C/P of 33.88 and C/CP of 38.93. Moreover, the United States also had the highest *h*-index (*h*) and *g*-index (*g*) of 162 and 292, respectively. This implies that there were 162 documents that have been cited 162 times or more while there were also 292 documents with a total citation of 85,264. Even though China had the second highest contribution in terms of the TP value, China had small C/P and C/CP values of only 13.72 and 16.60, respectively, which lagged behind Japan, England, Germany, Poland, France, and Taiwan. Table 4 lists the top 10 countries that contribute to the GaN literature.

Authors may collaborate with researchers across countries to produce better quality publications from impactful research for greater insights. Scientific collaboration, which is an intellectual cooperation, allows for knowledge, resource, and technology sharing among researchers in different regions. The synergy from these collaborations can be studied with a co-authorship analysis using VOSviewer software version 1.6.18 [90]. In the country co-authorship network diagram, the node size is proportional to the number of co-authored documents with other countries. When a country has high collaboration with other countries, the node size of that country will be large [91]. The color indicates the clustering of the node [90]. The thickness of the node between two countries signals the link strength between them. Link strength is explained by the number of documents co-authored by researchers in two countries, while the total link strength denotes the strength of a country’s collaborations with other countries [91].

**Figure 3:**
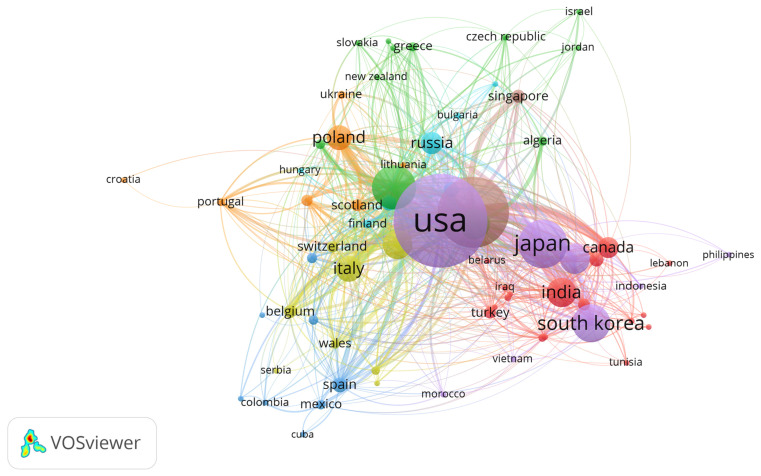
Country co-authorship network.

Table 5 presents the top 10 countries with the most co-authorships with other countries. The United States had 4685 publications with a TC of 158,750 and the highest total link strength of 1343. China, with 2808 publications and 38,518 TC, had the second highest total link strength of 749. Germany had the third highest total link strength of 667 with 1314 publications and 26,899 TC. The other countries with high total link strengths were England (476), Japan (456), France (448), Italy (357), South Korea (317), Poland (296), and Canada (205). Figure 3 depicts the country co-authorship network in GaN publications. The United States has the largest node because of its high total link strength. The highest link strength of 245 was observed between the United States and China as the line between these two countries is the thickest. The second highest link strength was between the United States and South Korea with a link strength of 131, followed by the link strength between the United States and Japan with a link strength of 121.

There are eight clusters in total. 19 countries, including Bangladesh, Belarus, Canada, Egypt, India, Iran, Iraq, Kazakhstan, Lebanon, Malaysia, Nigeria, Pakistan, Saudi Arabia, Thailand, Tunisia, Turkey, and the United Arab Emirates are in a similar cluster (red). The second cluster (green) consists of Algeria, Austria, Czech Republic, France, Germany, Greece, Israel, Jordan, Moldova, New Zealand, Romania, and Slovakia. The third cluster (blue) consists of Argentina, Brazil, Columbia, Cuba, Denmark, Mexico, South Africa, and Spain. The fourth cluster (yellow) is made up of countries such as Belgium, England, Ireland, Italy, North Ireland, Serbia, Switzerland, and Wales. The fifth cluster (purple) includes countries such as Indonesia, Japan, Morocco, Philippines, South Korea, Taiwan, the United States, and Vietnam. Croatia, Lithuania, the Netherlands, Poland, Portugal, Scotland, and Ukraine make up another cluster (light blue). The brown cluster consists of China and Singapore.

### 4.4. Source Title

There are about 210 source titles that have published papers related to GaN. Table 6 shows the top 10 source titles that have published GaN documents. The journal impact factor (JIF) computed by Clarivate shows the annual average number of citations of papers published in the previous two years in a journal [92]. The journal citation indicator (JCI) is the mean category normalized citation impact (CNCI) for all publications in a journal in the past three years. For the JCI 2021, the analysis is from 2018 to 2020. A JCI of 1.00 reflects average citation impact while values greater than 1.00 are higher than the average citation impact. On the other hand, a JCI of below 1.00 indicates a below average citation impact [93]. The CiteScore calculates the citations received in a year over the number of indexed publications in the past three years. The CiteScore 2021 shows the number of citations received in 2021 to the number of indexed publications from year 2018 to 2020. The SCImago journal rank (SJR) measures the impact of the journals by considering the number of citations and the performances of the cited journals. The source normalized impact per paper (SNIP), which is used to show the journal impact, is presented in Table 6 [94]. All indexes are in 2021. *Journal of Crystal Growth* published 626 papers, followed by *Applied Physics Letters* (624), *Journal of Applied Physics* (506), *IEEE Transactions on Electron Devices* (339), *Proceedings of SPIE* (312), *Physical Review B* (205), *IEEE Access* (201), *IEEE Electron Device Letters* (194), *Thin Solid Films* (193) and *Materials Research Society Symposium Proceedings* (187).

### 4.5. Most Cited Publications

Table 7 presents the top 10 most cited GaN publications. The most cited document “One-dimensional nanostructures: synthesis, characterization, and applications” by Xia et al. [87] received 8010 citations. This paper presented an overview on a variety of chemical methods that have been developed for generating nanostructures with 1D morphologies. The second most cited paper “Candela-class high-brightness InGaN/AIGaN double-heterostructure blue-light-emitting diodes” by Nakamura et al. [62] received 3307 citations. Candela-class high brightness InGaN/AIGaN DH blue LEDs with the luminous intensity were fabricated for the first time. The third most cited paper by Strite and Morkoc [95] presented the performance of several device structures that have been demonstrated in GaN material. Bernardini and Fiorentini [96] studied the spontaneous polarization, piezoelectric constants, and dynamical charges of the III-V nitride semiconductors AIN, GaN, and InN. The following most cited paper by Morkoc et al. [97] discussed the device-oriented research and applications of SiC, GaN, and ZnSe.

The sixth most cited paper by De Walle and Neugebauer [98] presented the state-of-the-art computational methodology for determining the structure and energetics of point defects and impurities in semiconductors as well as examples of defects and impurities in nitride semiconductors. The seventh most cited paper by Vurgaftman and Meyer [88] presented a compilation of band parameters for all of the nitrogen-containing III-V semiconductors. The following most cited paper by Ambacher et al. [99] investigated the formation of 2DEGs at the interfaces of pseudomorphic wurtzite and heterostructures involving GaN. Mueller et al. [100] mentioned that GaN-based materials enable light emission at blue and ultraviolet wavelengths. An asymmetric metallization scheme was adopted to break the mirror symmetry of the internal electric-field profile in conventional transistor channels, which allows for efficient photo detection. Amano et al. [101] mentioned that the quality of GaN thin films grown by MOVPE using AlN buffer layers is shown to be excellent in terms of morphological, crystalline, and optical properties.

### 4.6. Keyword Analysis

**Figure 4:**
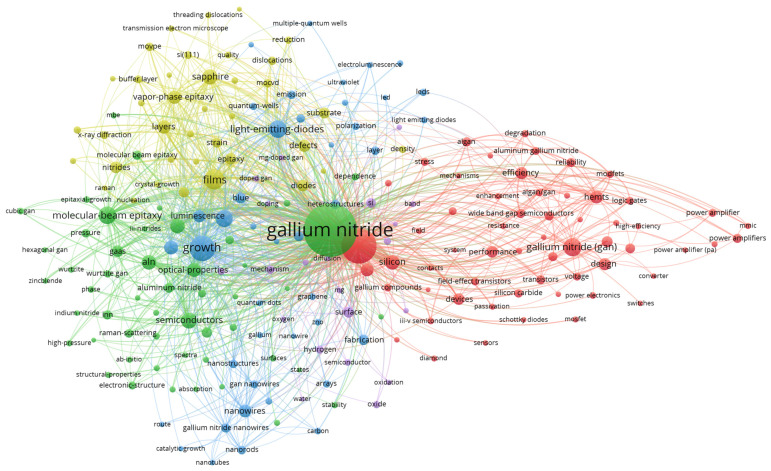
Keyword co-occurrence map.

The keyword co-occurrence map of VOSviewer studies the connections among the keywords. An advantage of the keyword co-occurrence map is that it allows researchers to identify key concepts and how these key concepts are connected to form sub-domains that may be the hotspots of research [102]. Table 8 displays the top 20 indexed keywords with the respective total link strengths. The keyword “gallium nitride”, with 5881 TP has the highest total link strength of 19,651. This implies that “gallium nitride” appeared the most with other keywords. GaN (8905) and growth (6233) also have high total link strengths. Figure 4 depicts the keyword co-occurrence map of GaN publications. The keyword “gallium nitride” most often appeared with “GaN” because it has the thickest line and highest link strength of 1018. “Gallium nitride” also has a high link strength with “growth” (753) and “films” (505).

From Figure 4, the keywords are grouped into five clusters made up of red, green, blue, yellow, and purple colors. The first cluster (red) has 59 keywords such as AlGaN, breakdown voltage, conductivity, current collapse, efficiency, electron mobility transistor, field effect transistors, high electron mobility transistors (HEMTs), III-V semiconductors, logic gates, modulation-doped field-effect transistor (MODFETs), metal oxide semiconductor field effect transistor (MOSFET), ohmic contacts, performance, power amplifier, Schottky diodes, sensors, switches, silicon, silicon carbide, and wide band semiconductors. The green cluster consists of the keywords of aluminium nitride, ammonia, crystals, dynamics, electronic properties, epitaxial growth, GaAs, gallium nitride, hexagonal GaN, high pressure, III-nitrides, indium nitride, molecular beam epitaxy, native defects, optical properties, photons, Raman scattering, spectroscopy, temperature dependence, thin films, wurtzite, and zinc blende. The blue cluster has the keywords carbon, catalytic growth, chemical vapor deposition, electroluminescence, emission, fabrication, gallium nitride nanowires, graphene, heterostructures, indium gallium nitride, light emitting diodes, luminescence, nanoparticles, nanostructures, nanotubes, photoluminescence, polarization, quantum dots, quantum wells, and ultraviolet. The fourth cluster (yellow) has the keywords buffer layer, cathodoluminescence, density, diodes, dislocations, hydride vapor phase epitaxy (HVPE), laser diodes, metal-organic chemical vapor deposition (MOCVD), morphology, metalorganic vapor phase epitaxy (MOVPE), nucleation, quality, reduction, sapphire, strain, threading dislocations and X-ray diffraction. The final cluster (purple) consists of activation, bond, doped GaN, doping, hydrogen, ion implantation, Mg-doped GaN, *n*-type GaN, oxidation, *p*-type GaN, and yellow luminescence.

**Figure 5:**
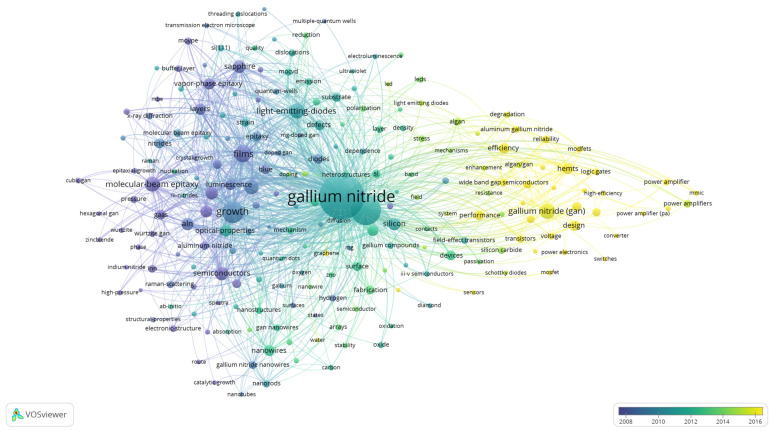
Overlay visualization map.

The trend of the GaN publication can be viewed with the overlay visualization map. In this map, the node color reflects the period the documents with the keyword was published. Darker colors imply that the key concepts (sub-domains) have been a long focus in the GaN research [103]. Figure 5 illustrates the overlay visualization map of GaN publications. The keywords in yellow are the recent focus of researchers. They include aluminium gallium nitride, HEMTs, wide band semiconductors, logic gates, power amplifier, power electronics, Schottky diodes, MOSFET, sensors, switches, converter, high efficiency, and MODFETs. This shows that researchers are paying a lot of attention in applying GaN in next generation devices. Due to its high efficiency, power density, carrier mobility in two-dimensional electron gas channels and critical electric fields, GaN is increasingly being studied for sensors, switches, and power electronics to handle high-voltage operations [104].

### 4.7. Citation Metrics

Citation metrics of the 15,634 documents on GaN publications from 1970 to 2023 as of 9 December 2022 have been extracted from Harzing’s Publish or Perish tool and tabulated in Table 9. Based on the 15,634 documents, 373,603 total citations have been received with the average of 23.90 citations per paper and an *h*-index of 188.

## 5. Conclusions

This paper presents a bibliometric analysis of the scientific literature of GaN listed in the internationally recognized Web of Science database from 1970 to 2023 as of 9 December 2022. The first three papers indexed in 1970 are titled “Optical studies of the photons and electrons in gallium nitride” by Manchon et al. [81], “Preparation of single phase gallium nitride from single crystal gallium arsenide” by Isherwood and Wickenden [82], and “Gallium nitride formed by vapour deposition and by conversion from gallium arsenide” by Faulkner et al. [83]. The largest production of GaN publications were found in 2020 with 1177 total documents. The highest cited document is “One-dimensional nanostructures: synthesis, characterization, and applications” by Xia et al. [88] which has received 8010 citations since its publication and indexing in 2003.

The scientific literature of GaN are mostly articles (67.16%) and proceeding papers (29.70%). Publication has been largely centered in the United States with 4685 total documents, 158,750 total citations, 33.88 citations per paper, and 38.93 citations per cited paper. The top source title that publishes GaN papers is the *Journal of Crystal Growth* published by Elsevier with TP of 626, impact factor of 1.830, citation indicator of 0.51, CiteScore of 3.5, and *h*-index of 155.

The country co-authorship network found that the United States has the largest total link strength of 1343, which implies that the United States is actively collaborating with other countries on GaN research areas. The highest link strength (245) is found between China and the United States. The keyword co-occurrence map is represented by five clusters. The first cluster is of high importance as the first cluster is also having the darkest color in the overlay visualization map, which implies that the first cluster has long been a focus in GaN research.

It is important to note that even though ongoing research has been performed on the thermostability and electronic properties of GaN, there is an increasing concentration on the application of GaN in next generation devices, especially for satellites and 5G technologies and beyond. GaN is often studied for its applications in the areas of power electronics, power amplifier, wide band gap semiconductors, sensors, and switches that have high potential to drive digitization and further industrialization. The outstanding properties of GaN as a semiconductor make it attractive to be applied in these areas for future advancement.

## Figures and Tables

**Table 1:** Document types of GaN publications.

Document	Frequency	Percentage (%)
Article	11,559	67.16
Proceeding Paper	5112	29.70
Review Article	311	1.80
Book Chapter	86	0.50
Early Access	59	0.34
News Item	40	0.23
Editorial Material	39	0.23
Book	5	0.03
Book Review	1	0.01
Total	17,212	100.00

**Table 2:** Production growth of GaN documents.

Year	TP	Percentage	Cumulative Percentage	NCP	TC	C/P	C/CP	*h*	*g*
1970	3	0.02	0.02	3	216	43.2	72	3	3
1971	5	0.03	0.05	5	375	75	75	5	5
1973	3	0.02	0.07	3	282	94	94	2	3
1974	11	0.07	0.14	11	279	25.36	25.36	11	11
1975	3	0.02	0.16	3	154	51.33	51.33	3	3
1976	3	0.02	0.18	3	21	7	7	2	3
1977	2	0.01	0.19	2	8	4	4	2	2
1978	7	0.04	0.24	6	250	35.71	41.67	6	7
1979	4	0.03	0.26	3	27	6.75	9	2	4
1980	7	0.04	0.31	6	209	29.86	34.83	5	7
1981	4	0.03	0.33	3	62	15.5	20.67	3	4
1982	2	0.01	0.35	2	4	2	2	1	2
1983	4	0.03	0.37	3	32	8	10.67	2	4
1984	2	0.01	0.38	2	4	2	2	2	2
1985	1	0.01	0.39	0	0	0	0	0	0
1986	6	0.04	0.43	6	1913	318.83	318.83	4	5
1987	1	0.01	0.43	1	1	1	1	1	1
1988	3	0.02	0.45	3	270	90	90	3	3
1989	1	0.01	0.46	1	332	332	332	1	1
1990	8	0.05	0.51	7	333	41.63	47.57	6	8
1991	16	0.10	0.61	16	1074	67.13	67.13	12	16
1992	19	0.12	0.74	17	4775	251.32	280.88	14	18
1993	47	0.30	1.04	45	4051	86.19	90.02	30	47
1994	64	0.41	1.45	63	10,278	160.59	163.14	39	62
1995	108	0.69	2.14	100	5944	55.04	59.44	43	75
1996	246	1.57	3.71	230	12,986	52.79	56.46	52	104
1997	322	2.06	5.77	307	16,460	51.12	53.62	54	103
1998	356	2.28	8.05	330	14,231	39.97	43.12	55	101
1999	426	2.72	10.77	407	15,534	36.46	38.17	54	90
2000	407	2.60	13.37	378	12,724	31.26	33.66	58	87
2001	351	2.25	15.62	334	11,000	31.34	32.93	51	83
2002	361	2.31	17.93	339	15,363	42.56	45.32	53	109
2003	372	2.38	20.31	352	22,034	59.23	62.6	54	131
2004	333	2.13	22.44	307	12,642	37.96	41.18	49	82
2005	393	2.51	24.95	363	12,018	30.58	33.11	51	89
2006	467	2.99	27.94	435	12,464	26.69	28.65	50	84
2007	464	2.97	30.91	422	10,877	23.44	25.77	49	74
2008	511	3.27	34.18	470	12,958	25.36	27.57	50	84
2009	453	2.90	37.07	417	11,268	24.87	27.02	43	83
2010	416	2.66	39.73	377	10,718	25.76	28.43	41	84
2011	496	3.17	42.91	462	10,414	21	22.54	48	72
2012	573	3.67	46.57	516	13,137	22.93	25.46	46	86
2013	592	3.79	50.36	532	12,529	21.16	23.55	52	80
2014	698	4.46	54.82	630	13,317	19.08	21.14	45	77
2015	684	4.38	59.20	615	12,743	18.63	20.72	47	74
2016	786	5.03	64.23	708	13,647	17.36	19.28	50	73
2017	838	5.36	69.59	747	14,336	17.11	19.19	50	73
2018	838	5.36	74.95	731	14,406	17.19	19.71	51	78
2019	890	5.69	80.64	758	11,970	13.45	15.79	46	65
2020	1177	7.53	88.17	1002	13,654	11.6	13.63	44	61
2021	1091	6.98	95.15	825	7502	6.88	9.09	46	13
2022	742	4.75	99.89	328	1747	2.35	5.33	19	28
2023	17	0.11	100.00	2	30	1.76	15	2	5
TOTAL	15,634	100			373,603				

**Table 3:** Subject areas of GaN publications.

Subject Area	TP
Physics	8250
Engineering	6634
Materials Science	5197
Chemistry	1607
Science Technology and Other Topics	1528
Optics	1337
Crystallography	827
Telecommunications	805
Computer Science	762
Energy Fuels	716
Instruments Instrumentation	446
Metallurgy Metallurgical Engineering	244
Electrochemistry	221
Nuclear Science Technology	179
Automation Control Systems	171
Imaging Science and Photographic Technology	113
Remote Sensing	105
Thermodynamics	85
Geochemistry/Geophysics	74
Mechanics	58
Microscopy	54

**Table 4:** Top 10 contribution by country.

Country	TP	NCP	TC	C/P	C/CP	*h*	*g*
United States	4685	4078	158,750	33.88	38.93	162	292
China	2808	2320	38,518	13.72	16.60	72	130
Japan	1531	1315	34,338	22.43	26.11	79	146
Germany	1314	1137	26,899	20.47	23.66	77	124
South Korea	971	857	13,798	14.21	16.10	50	85
France	724	597	13,356	18.45	22.37	50	94
India	695	513	6668	9.59	13.00	36	56
Taiwan	686	565	11,494	16.76	20.34	47	86
England	659	567	14,438	21.91	25.46	50	100
Poland	550	466	10,332	18.79	22.17	45	84

**Table 5:** Country co-authorship in GaN publications.

Country	Document	Total Link Strength
United States	4685	1343
China	2808	749
Germany	1314	667
England	659	476
Japan	1531	456
France	724	448
Italy	539	357
South Korea	971	317
Poland	550	296
Canada	370	205

**Table 6:** Source titles in GaN publications.

Source Title	TP	%	TC	Publisher	JIF	JCI	Cite Score	SJR	SNIP	*h*
*Journal of Crystal Growth*	626	4.01	14,171	Elsevier	1.830	0.51	3.5	0.43	0.839	155
*Applied Physics Letters*	624	3.99	28,938	AIP Publishing	3.971	0.80	6.6	1.025	1.119	452
*Journal of Applied Physics*	506	3.24	26,238	AIP Publishing	2.877	0.57	4.7	0.668	0.964	331
*IEEE Transactions on Electron Devices*	339	2.17	7831	IEEE-Institute of Electrical and Electronics Engineers Inc.	3.221	0.75	5.3	0.695	1.364	191
*Proceedings of SPIE*	312	2.00	813	SPIE	N/A	N/A	N/A	0.184	N/A	179
*Physical Review B*	205	1.31	18,086	American Physical Society	3.908	0.76	N/A	1.537	N/A	460
*IEEE Access*	201	1.29	1653	IEEE-Institute of Electrical and Electronics Engineers Inc.	3.476	0.93	6.7	0.927	1.326	158
*IEEE Electron Device Letters*	194	1.24	5657	IEEE-Institute of Electrical and Electronics Engineers Inc.	4.816	1.25	8.5	1.13	1.649	159
*Thin Solid Films*	193	1.24	2240	Elsevier Science SA	2.378	0.42	4.3	0.468	0.772	199
*Materials Research Society Symposium Proceedings*	187	1.20	945	Materials Research Society	N/A	N/A	N/A	N/A	N/A	60

**Table 7:** Top 10 most cited GaN publications.

Title	Year	TC	Source Title
One-dimensional nanostructures: synthesis, characterization, and applications [87]	2003	8010	*Advanced Materials*
Candela-class high-brightness InGaN/AlGaN double-heterostructure blue-light-emitting diodes [62]	1994	3307	*Applied Physics Letters*
GaN, AlN and InN: A review [95]	1992	2636	*Journal of Vacuum Science & Technology B*
Spontaneous polarization and piezoelectric constants of III-V nitride [96]	1997	2500	*Physical Review B*
Large band gap SiC, IIIV nitride, and IIVI ZnSe-based semiconductor device technologies [97]	1994	2452	*Journal of Applied Physics*
First-principles calculations for defects and impurities: Applications to III-nitrides [98]	2004	2396	*Journal of Applied Physics*
Band parameters for nitrogen-containing semiconductors [88]	2003	2312	*Journal of Applied Physics*
Two-dimensional electron gases induced by spontaneous and piezoelectric polarization charges in N- and Ga-face AlGaN/GaN heterostructures [99]	1999	2230	*Journal of Applied Physics*
Graphene photodetectors for high-speed optical communications [100]	2010	1860	*Nature Photonics*
Metalorganic vapor phase epitaxial growth of a high quality GaN film using an AlN buffer layer [101]	1986	1856	*Applied Physics Letters*

**Table 8:** Indexed keywords.

Keyword	TP	Total Link Strength
Gallium Nitride	5881	19,651
GaN	2537	8905
Growth	1376	6233
Films	833	3981
Molecular Beam Epitaxy	642	3146
Light Emitting Diodes	677	3040
Photoluminescence	597	2901
Sapphire	507	2630
Semiconductors	521	2307
AlN	454	2253
Thin Films	448	2127
Optical Properties	424	2115
Silicon	446	2063
Chemical Vapor Deposition	406	2017
Vapor Phase Epitaxy	391	1932
Layers	368	1769
Temperature	362	1693
Nanowires	350	1629
HEMTs	359	1556
Defects	330	1549

**Table 9:** Citation metrics.

Items	Metrics
Extraction Date	9 December 2022
Number of Documents	15,634
Total Citations	373,603
Period of Analysis	52
Citations per Year	6806.04
Citations per Paper	23.90
Citations per Author	93,337.41
Papers per Author	4045.49
Authors per Paper	5.25
*h*-index	188
*g*-index	310

## Data Availability

The data presented in this study are available on request from the corresponding author.
